# Citation: Tight Junction Protein Expression-Inducing Probiotics Alleviate TNBS-Induced Cognitive Impairment with Colitis in Mice

**DOI:** 10.3390/nu14142975

**Published:** 2022-07-20

**Authors:** Xiao-Yang Ma, Young-Hoo Son, Jong-Wook Yoo, Min-Kyung Joo, Dong-Hyun Kim

**Affiliations:** Neurobiota Research Center, College of Pharmacy, Kyung Hee University, 26, Kyungheedae-ro, Dongdaemun-gu, Seoul 02447, Korea; xiaoyangma12@gmail.com (X.-Y.M.); bitfl@naver.com (Y.-H.S.); hooit96@naver.com (J.-W.Y.); mkiti1727@gmail.com (M.-K.J.)

**Keywords:** tight junction protein, cognitive impairment, gut inflammation, *Lactobacillus rhamnosus*, *Lactobacoccus lactis*, *Bifidobacterium longum*

## Abstract

A leaky gut is closely connected with systemic inflammation and psychiatric disorder. The rectal injection of 2,4,6-trinitrobenzenesulfonic acid (TNBS) induces gut inflammation and cognitive function in mice. Therefore, we selected *Bifidobacterium longum* NK219, *Lactococcus lactis* NK209, and *Lactobacillus rhamnosus* NK210, which induced claudin-1 expression in TNBS- or lipopolysaccharide (LPS)-stimulated Caco-2 cells, from the fecal bacteria collection of humans and investigated their effects on cognitive function and systemic inflammatory immune response in TNBS-treated mice. The intrarectal injection of TNBS increased cognitive impairment-like behaviors in the novel object recognition and Y-maze tests, TNF-α, IL-1β, and IL-17 expression in the hippocampus and colon, and LPS level in the blood and feces, while the expression of hippocampal claudin-5 and colonic claudin-1 decreased. Oral administration of NK209, NK210, and NK219 singly or together decreased TNBS-impaired cognitive behaviors, TNF-α and IL-1β expression, NF-κB^+^Iba1^+^ cell and LPS^+^Iba1^+^ cell numbers in the hippocampus, and LPS level in the blood and feces, whereas BDNF^+^NeuN^+^ cell and claudin-5^+^ cell numbers and IL-10 expression increased. Furthermore, they suppressed TNBS-induced colon shortening and colonic TNF-α and IL-1β expression, while colonic IL-10 expression and mucin protein-2^+^ cell and claudin-1^+^ cell numbers expression increased. Of these, NK219 most strongly alleviated cognitive impairment and colitis. They additively alleviated cognitive impairment with colitis. Based on these findings, NK209, NK210, NK219, and their combinations may alleviate cognitive impairment with systemic inflammation by suppressing the absorption of gut bacterial products including LPS into the blood through the suppression of gut bacterial LPS production and alleviation of a leaky gut by increasing gut tight junction proteins and mucin-2 expression.

## 1. Introduction

A leaky gut, a breakdown of the gut physical barrier, increases the gut permeability with systemic inflammation [1,2]. The gut permeability is mainly dependent on the tight junction protein expression in the gastrointestinal tract, such as occludin, claudins, and zonula occludens, and mucus layer, such as mucin-2 [1,2]. The invasion of bacteria into the body causes a variety of inflammation such as colitis, chorioamnionitis, and cystitis, which suppresses the tight junction protein expression [3,4,5]. The suppression of tight junction protein and/or mucus layer expression by gut inflammation increases the absorption of gut bacterial antigens such as lipopolysaccharides (LPS) from the gastrointestinal tract into the blood, resulting in cognitive impairment with systemic inflammation [6,7,8]. 

The intrarectal injection of 2,4,6-trinitrobenzenesulfonic acid (TNBS) impairs cognitive function and suppresses tight junction protein expression, resulting in a leaky gut (LPS-absorbable colitis) [9,10]. Therefore, maintaining the gut physical barrier can be useful for the treatment of cognitive impairment. 

Probiotics including Lactobacilli and Bifidobacteria are microbes that have various health benefits [11]. They alleviate gut microbiota dysbiosis [11,12], immune imbalance [12], gut and systemic inflammation [13,14], depression [15], and cognitive impairment [16]. *Lactobacillus plantarum* C29, which was isolated from a fermented food, suppresses memory impairment in aged and 5XFAD mice and its contained supplement increases cognitive function in individuals with middle cognitive impairment [17,18,19]. *Lactobacillus johnsonii*, whose population is decreased in TNBS-treated mice, alleviates memory damage with gut inflammation in mice [9]. *Bifidobacterium longum* NK46 increases cognitive function in 5XFAD-transgenic mice by modifying gut microbiota and inhibiting gut bacteria LPS production [20]. *Lactobacillus reuteri* NK33 and *Bifidobacterium adolescentis* NK98 mitigate depression with gut inflammation by modifying gut microbiota [21]. NVP1704, a mixture of *Lactobacillus reuteri* NK33 and *Bifidobacterium adolescentis* NK98, mitigates depressive symptoms in individuals with depression and insomnia [22]. These findings suggest that gut permeability-suppressing probiotics may mitigate colitis and cognitive impairment in vivo. Nevertheless, understanding whether tight junction protein expression-inducing gut bacteria can simultaneously mitigate colitis and cognitive impairment is not sufficient. 

Therefore, we selected *Lactococcus lactis* NK209 (Ll), *Lactobacillus rhamnosus* NK210 (Lr), and *Bifidobacterium longum* NK219 (Bl), which increased claudin-1 expression in LPS- or TNBS-treated Caco-2 cells, from the fecal bacteria collection of humans and investigated their effects on TNBS-induced leaky gut and cognitive impairment in mice. 

## 2. Materials and Methods

### 2.1. Materials

LPS, TNBS, 4′,6-diamidino-2-phenylindole dilactate (DAPI), and DMEM were purchased from Sigma (St Louis, MO, USA). An antibody for NF-κB was purchased from Cell Signaling Technology (Danvers, MA, USA). Alexa Fluor 488 and Alexa Fluor 594 were purchased from Invitrogen (Carbsband, CA, USA). Enzyme-linked immunosorbent assay (ELISA) kits for TNF-α, IL-1β, IL-10, IL-17, and myeloperoxidase (MPO) were purchased from R&D (Minneapolis, MN, USA).

### 2.2. Culture of Gut Bacteria

NK209, NK210, and NK219 were cultured in commercial media for probiotics including MRS broth (BD, Franklin Lakes, NJ, USA), centrifuged at 5000× *g* and at 4 °C for 20 min, and washed with saline [23,24]. The collected cells were used for experiments.

### 2.3. Culture of Caco-2 Cells

Caco-2 cells were cultured in DMEM containing 10% fetal bovine serum and 1% antibiotic-antimycotic at 37 °C, as previously reported [24]. The cells (1 × 10^5^ cells/well) were incubated with probiotics (1 × 10^5^ CFU/mL) in the absence or presence of TNBS or LPS in 12-well plate for 22 h and washed twice. The cells were collected and lysed in RIPA lysis buffer containing 1% phosphatase inhibitor cocktail and 1% protease inhibitor cocktail on ice. The claudin-1 expression level in the supernatant was measured, assessed by immunoblotting.

### 2.4. Animals

Male C57BL/6 mice (7-weeks old, 19–21 g) were purchased from Koatech Inc. (Seoul, Korea) and maintained under the controlled condition (temperature, 20–22 °C; humidity, 40–60% humidity; light/dark cycle, 12 h) and fed standard laboratory chow and water ad libitum. Mice were acclimatized for 1 week before experiments. All animal experiments were approved by the Kyung Hee University Institutional Animal Care and Use Committee of (IACUC No., KUASP(SE)-21451) and performed according to the Guide for University Laboratory Animals Care and Usage. 

### 2.5. Preparation of Mice with Colitis

For the preparation of mice with colitis, mice were anesthetized with 1.5% isofurane and 0.1 mL of 2.5% (*w*/*v*) TNBS solution (diluted in 50% ethanol) was intrarectally injected into the colon. Mice were vertically held for 30 s [9]. Probiotics (TNBS, vehicle alone; Ll, 1 × 10^9^ CFU/mouse of *Lactococcus lactis* NK209; Lr, 1 × 10^9^ CFU/mouse of *Lactobacillus rhamnosus* NK210; Bl, 1 × 10^9^ CFU/mouse of *Bifidobacterium longum* NK219; LrLl, 1 × 10^9^ CFU/mouse of NK209 and NK210 (1:1) mix; LlBl, 1 × 10^9^ CFU/mouse of NK210 and NK219 (4:1) mixture; LrBl, 1 × 10^9^ CFU/mouse of NK209 and NK219 (4:1) mix; LLB, 1 × 10^9^ CFU/mouse of NK209, NK210, and NK219 (2:2:1) mix) were orally gavaged daily for 5 days from 20 h after the final TNBS injection. Normal control mice were treated with 1% maltose (vehicle) alone instead of TNBS and test agents. 

Cognitive behaviors were evaluated 2 h after the final probiotic treatment in the Y-maze task (YMT) and novel object recognition task (NORT). Mice were euthanized by cervical dislocation under the inhalation of CO_2_ 20 h after the behavioral tasks. Colon and brain tissues were collected for the analyses of ELISA and immunoblotting and stored at −80 °C. 

### 2.6. Assessment of Cognitive Behavior Tasks

The YMT was performed in a three-arm horizontal maze (40 cm (length), 3 cm (width) and 12 cm (height)) for 8 min, as previously reported [19,23]. The spontaneous alteration (%) was described as a spontaneous to possible alternation ratio. The NORT was performed in a black rectangular open field maze (45 × 45 × 45 cm) for 10 min, as previously reported [17,19]. The exploration (%) was described as the new object-touching time to the old and neo-object-touching time ratio. 

### 2.7. Measurement of LPS Concentration

The blood endotoxin level was assayed using the diazo-coupled limulus amoebocyte lysate (LAL) assay kit (Cape Cod Inc., East Falmouth, MA, USA) [25]. 

### 2.8. ELISA Assay and Immunblotting

The tissues were lysed in RIPA lysis buffer and centrifuged (10,000× *g*, 4 °C, 10 min) [25]. In the supernatant, biomarkers were assayed using commercial ELISA kits for MPO (R&D, DY3667, Minneapolis, MN, USA), BDNF (R&D, DY248), IL-1β (R&D, DY401), IL-10 (R&D, DY417), IL-17 (Invitrogen, 88-7371-88), and TNF-α (R&D, DY410).

### 2.9. Immunofluorescence Assay

Mice were transcardially perfused, as previously reported [25,26]. Their brain and colon tissues were post-fixed, cytoprotected, frozen, sectioned, and washed [19,26]. The washed section was blocked with serum, incubated with primary antibodies for BDNF (1:200, Santa Cruz Biotechnology, #SC-65513, canonical nerve growth factor), NeuN (1:200, Millipore, #MAB377, a neuronal nuclear antigen), NF-κB (1:100, Cell Signaling Technology, #3033S), Iba1 (1:200, Thermo Fisher Scientific, #PA5-27436, a microglial protein), LPS (1:200, Abcam, #ab35654, a Gram-negative bacterial endotoxin), MUC2 (1:500, Abcam, #ab272692), claudin-1 (1:200, Santa Cruz, #G0119, an intestinal tight junction protein), claudin-5 (1:200, Millipore, ABT45, a brain tight junction protein), and/or CD11c (1:200, Abcam, #ab11029, an immune cell protein) overnight, washed, and incubated with the secondary antibodies conjugated with Alexa Fluor 594 (1:200, Invitrogen) or Alexa Fluor 488 (1:200, Invitrogen) for 2 h. The section was also stained with DAPI and photographed by a confocal laser microscope.

### 2.10. Statistical Analysis

All data are described as means ± standard deviation (SD). The significance (*p* < 0.05) was analyzed by a one-way analysis of variance, followed by Kruskal–Wallis test with Dunn’s post-hoc test, using GraphPad Prism 9 (GraphPad Software, Inc., San Diego, CA, USA).

## 3. Results

### 3.1. NK209, NK210, and NK219 Increased Claudin-1 Expression in TNBS-Stimulated Caco-2 Cells

**Figure 1:**
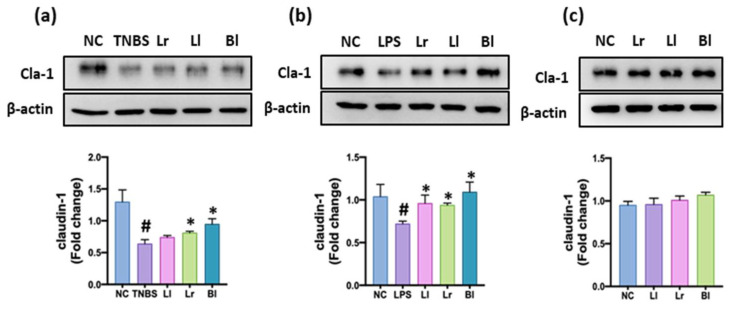
Effects of NK209 (Ll), NK210 (Lr), and NK219 (Bl) on the expression of claudin-1 in TNBS- or LPS-stimulated Caco-2 cells. Caco-2 cells were incubated with probiotics in the absence or presence of TNBS (**a**), LPS (**b**) or saline (**c**). Normal control (NC) was treated with saline instead of LPS or TNBS. Data were indicated as mean ± SD (*n* = 4). Data indicate mean ± SD. ^#^ *p* < 0.05 vs. NC group. * *p* < 0.05 vs. TNBS or LPS group.

Of the tested Lactobacilli and Bifidobacteria, NK209, NK210, and NK219 potently increased claudin-1 expression in TNBS-treated Caco-2 cells, whereas they did not affect claudin-1 expression in Caco-2 treated with vehicle saline alone (Figure 1). They also increased LPS-induced suppression of claudin-1 in Caco-2 cells. NK209, NK210, and NK219 were identified as *L. lactis*, *L. rhamnosus*, and *B. longum*, based on Gram staining, API kits, and 16S rRNA gene sequence.

### 3.2. NK209, NK210, and NK219 Mitigated TNBS-Induced Cognitive Impairment and Neuroinflammation in Mice

**Figure 2:**
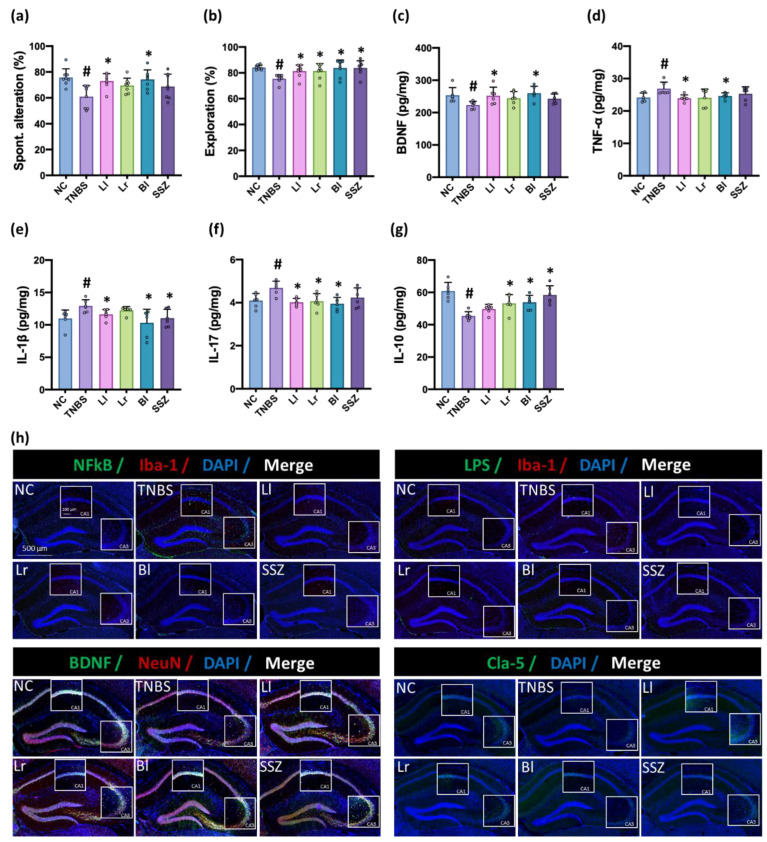
Effects of NK209 (Ll), NK210 (Lr), and NK219 (Bl) on TNBS-impaired cognitive function in mice. Effects on spontaneous alteration in YMT (**a**) and exploration in NORT (**b**). Effects on the expression of BDNF (**c**), TNF-α (**d**), IL-1β (**e**), IL-17 (**f**), and IL-10 (**g**) in the hippocampus, assessed by ELISA. (**h**) Effects on NF-κB^+^Iba1^+^. BDNF^+^NeuN^+^, LPS^+^Iba1^+^, and claudin (Cla)-5^+^ cell populations in the hippocampus, assessed by a confocal microscope. Probiotics (TNBS, vehicle; Ll, 1 × 10^9^ CFU/mouse/day of NK209; Lr, 1 × 10^9^ CFU/mouse/day of NK210; Bl, 1 × 10^9^ CFU/mouse/day of NK219; SSZ, 25 mg/kg of sulfasalazine) were orally gavaged daily for 5 days after intrarectal injection of TNBS. Normal control (NC) was treated with vehicle (1% maltose) instead of probiotics. Data indicate mean ± SD (*n* = 8). ^#^ *p* < 0.05 vs. NC. * *p* < 0.05 vs. TNBS.

The intrarectal injection of TNBS impaired cognitive function in the YMT and NORT to 80.4% (F(5,42) = 4.242, *p* = 0.0033) and 89.8% (F(5,42) = 3.6, *p* = 0.0073) of normal control mice, respectively, as previously reported [9] (Figure 2, Supplementary Figure S1). TNBS treatment increased TNF-α and IL-1β expression and NF-κB^+^Iba1^+^ cell and LPS^+^Iba1^+^ cell numbers in the hippocampus. Oral administration of NK209 (Ll), NK210 (Lr), NK219 (Bl), or sulfasalazine significantly recovered TNBS-impaired cognitive behaviors in the YMT to 96.3%, 91.8%, 98.0%, and 90.6% (F(5,42) = 4.242, *p* = 0.0033) of normal control mice, respectively, and in the NORT to 96.7%, 96.8%, 99.6%, and 99.7% (F(5,42) = 3.6, *p* = 0.0053) of normal control mice, respectively. They also decreased TNBS-induced TNF-α, IL-1β, and IL-17 expression and NF-κB^+^Iba1^+^ cell and LPS^+^Iba1^+^ cell numbers in the hippocampus and increased TNBS-suppressed BDNF and IL-10 expression and BDNF^+^NeuN^+^ cell and claudin-5^+^ cell numbers in the hippocampus. Overall, NK219 most potently alleviated TNBS-induced cognitive impairment-like behaviors and neuroinflammation. 

### 3.3. NK209, NK210, and NK219 Mitigated TNBS-Induced Gut Inflammation in Mice

**Figure 3:**
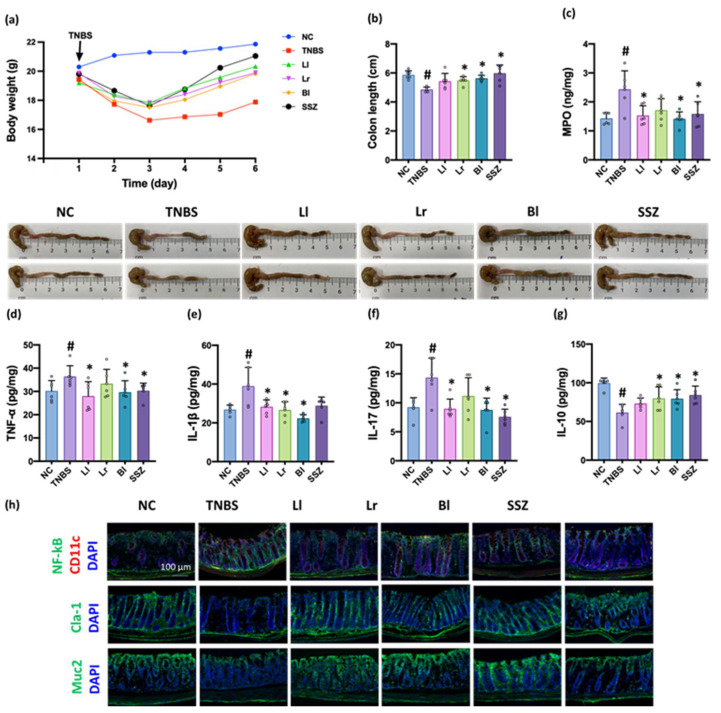
Effects of NK209, NK210, and NK219 on TNBS-induced colitis in mice. (**a**) Effects on bodyweight. (**b**) Effects on colon length. Effects on myeloperoxidase (MPO, (**c**)), TNF-α (**d**), IL-1β (**e**), IL-17 (**f**), and IL-10 expression (**g**) in the colon, assessed by ELISA, and NF-κB^+^CD11c^+^, claudin (Cla)-1^+^, and mucin (Muc)2^+^ cell populations (**h**), assessed by a confocal microscope. Probiotics (TNBS, TNBS alone; Ll, 1 × 10^9^ CFU/mouse/day of NK209; Lr, 1 × 10^9^ CFU/mouse/day of NK210; Bl, 1 × 10^9^ CFU/mouse/day of NK219; SSZ, 25 mg/kg of sulfasalazine) were orally gavaged daily for 5 days after intrarectal injection of TNBS. Normal control (NC) was treated with vehicle (1% maltose) instead of test agents. Data indicate mean ± SD (*n* = 6). ^#^ *p* < 0.05 vs. NC. * *p* < 0.05 vs. TNBS.

The intrarectal injection of TNBS shortened colon length, induced myeloperoxidase and TNF-α expression, increased NF-κB^+^CD11c^+^ cell population, and decreased IL-10 and mucin-2 expression in the colon (Figure 3, Supplementary Figure S2). However, oral administration of NK209 (Ll), NK210 (Lr), NK219 (Bl), or sulfasalazine (SSZ) inhibited TNBS-induced colon shortening, myeloperoxidase, TNF-α, IL-1β, and IL-17 expression, and NF-κB^+^CD11c^+^ cell population in the colon and increased TNBS-suppressed IL-10 expression and mucin-2^+^ cell and claudin-1^+^ cell numbers. They alleviated TNBS-induced gut inflammation. Of these, NK219 most potently alleviated TNBS-induced gut inflammation.

### 3.4. NK209, NK210, and NK219 Suppressed TNBS-Induced LPS Levels in the Blood and Feces

**Figure 4:**
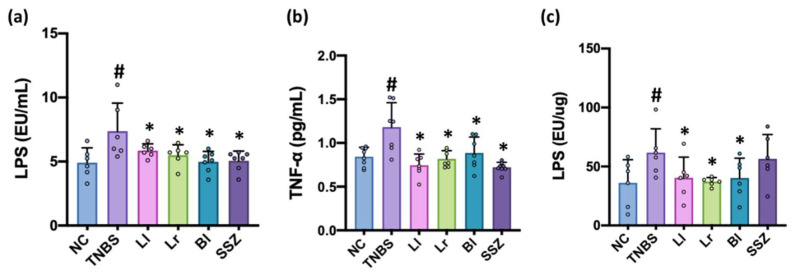
Effects of NK209, NK210, and NK219 on the levels of blood LPS (**a**) and TNF-α (**b**) and fecal LPS (**c**) in TNBS-treated mice. Probiotics (TNBS, TNBS alone; Ll, 1 × 10^9^ CFU/mouse/day of NK209; Lr, 1 × 10^9^ CFU/mouse/day of NK210; Bl, 1 × 10^9^ CFU/mouse/day of NK219; SSZ, 25 mg/kg of sulfasalazine) were orally gavaged daily for 5 days after TNBS treatment. Normal control (NC) was treated with vehicle instead of probiotics. Data indicate mean ± SD (*n* = 6). ^#^ *p* <0.05 vs. NC. * *p* < 0.05 vs. TNBS.

The intrarectal injection of TNBS increased LPS level in the blood and feces, as previously reported [7] (Figure 4, Supplementary Figure S3). Treatment with NK209, NK210, or NK219 significantly decreased TNBS-induced LPS and TNF-α levels in the blood and LPS level in the feces. 

### 3.5. The Combined Effects of NK209, NK210, and NK219 on TNBS-Induced Cognitive Impairment and Gut Inflammation in Mice

**Figure 5:**
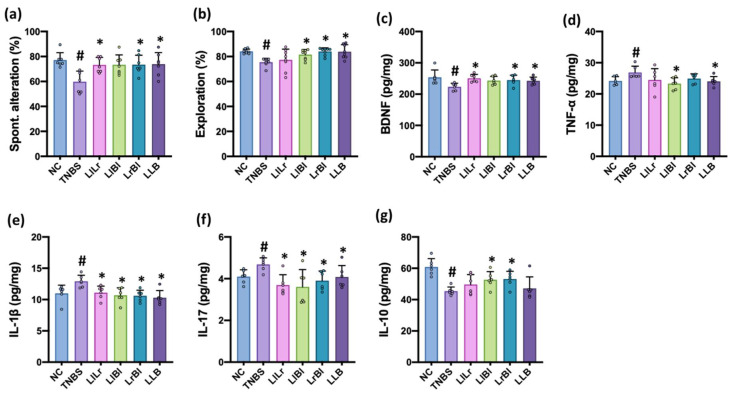

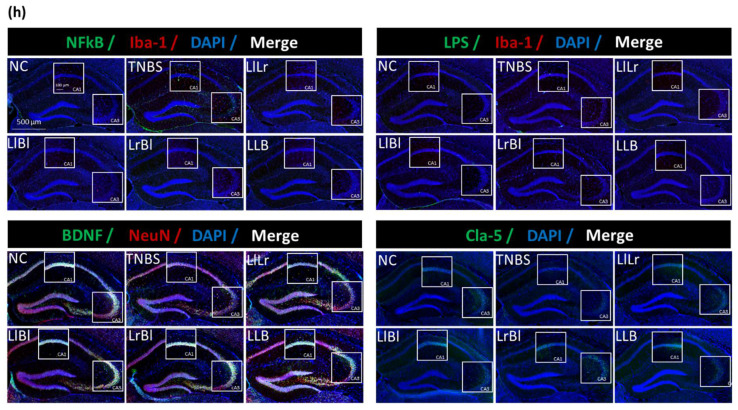
The combined effects of NK209, NK210, and NK219 on TNBS-impaired cognitive function in mice. Effects on spontaneous alteration in Y-maze test (**a**) and exploration in novel object recognition test (**b**). Effects on the expression of BDNF (**c**), TNF-α (**d**), IL-1β (**e**), IL-17 (**f**), and IL-10 (**g**) in the hippocampus. (**h**) Effects on NF-κB^+^Iba1^+^. BDNF^+^NeuN^+^, LPS^+^Iba1^+^, claudin (Cla)5^+^ cell populations in the hippocampus. Test agents [TNBS, TNBS alone; LlLr, 1 × 10^9^ CFU/mouse/day of NK209 and NK210 (1:1) mix; LlBl, 1 × 10^9^ CFU/mouse/day of NK209 and NK219 (4:1) mix; LrBl, 1 × 10^9^ CFU/mouse/day of NK210 and NK219 (4:1) mix; LLB, 1 × 10^9^ CFU/mouse/day of NK209, NK210, and NK219 (2:2:1) mix] were orally administered daily for 5 days after TNBS treatment. Normal control (NC) was treated with vehicle instead of test agents. Data indicate mean ± SD (*n* = 8). ^#^ *p* <0.05 vs. NC. * *p* < 0.05 vs. TNBS.

To understand the combined effects of NK209, NK210, and NK219, we orally administered LlLr (NK209 + NK210 [1:1] mix), LlBl (NK209 + 219 [4:1] mix), LrBl (NK210 + NK219 [4;1] mix), or LLB (NK209 + NK210 + NK219 [2:2:1] mix) and examined their effects on TNBS-impaired cognitive function and gut immune response in mice (Figure 5, Supplementary Figure S3). The intrarectal injection of TNBS impaired cognitive behaviors in the YMT and NORT and increased hippocampal TNF-α and IL-1β expression and NF-κB^+^Iba1^+^ cell and LPS^+^Iba1^+^ cell numbers. However, oral administration of LlLr, LlBl, LrBl, or LLB significantly recovered TNBS-impaired cognitive behaviors in the Y-maze task to 94.4%, 94.5%, 95.5%, and 95.3% [F(5,42) = 3.1, *p* = 0.0179] of normal control mice, respectively, and in the NOR task to 91.9%, 97.0%, 99.9%, and 99.8% [F(5,42) = 4.842, *p* = 0.0014] of normal control mice, respectively. They also reduced TNBS-induced TNF-α, IL-1β, and IL-17 expression and NF-κB^+^Iba1^+^ cell and LPS^+^Iba1^+^ cell numbers in the hippocampus and increased TNBS-suppressed BDNF and IL-10 expression and BDNF^+^NeuN^+^ cell and claudin-5^+^ cell numbers in the hippocampus. These combinations additively alleviated TNBS-induced cognitive impairment with neuroinflammation.

**Figure 6:**
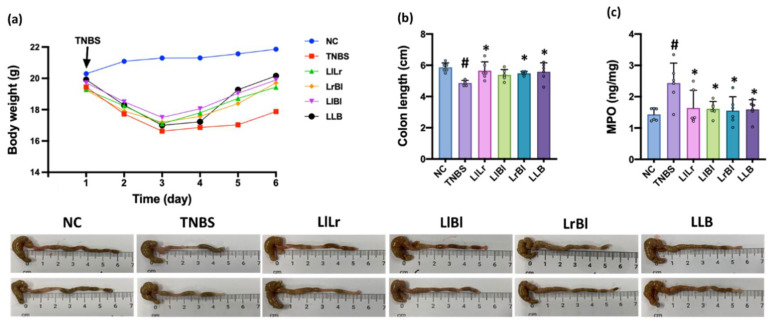

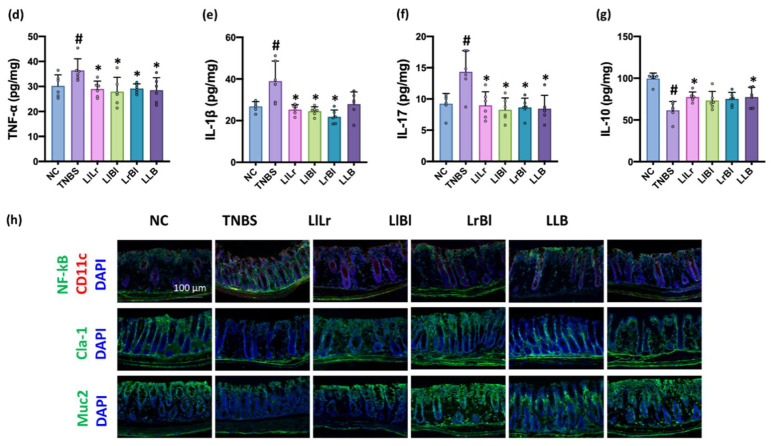
The combined effects of NK209, NK210, and NK219 on TNBS-induced colitis in mice. (**a**) Effects on bodyweight (**a**), colon length (**b**), myeloperoxidase (MPO, (**c**)), TNF-α (**d**), IL-1β (**e**), IL-17 (**f**), and IL-10 expression (**g**), and NF-κB^+^CD11c^+^, claudin (Cla)-1^+^, and mucin (Muc)2^+^ cell populations (**h**). Probiotics [TNBS, TNBS alone; LlLr, 1 × 10^9^ CFU/mouse/day of NK209 and NK210 (1:1) mix; LlBl, 1 × 10^9^ CFU/mouse/day of NK209 and NK219 (4:1) mix; LrBl, 1 × 10^9^ CFU/mouse/day of NK210 and NK219 (4:1) mix; LLB, 1 × 10^9^ CFU/mouse/day of NK209, NK210, and NK219 (2:2:1) mix] were orally administered daily for 5 days after TNBS treatment. Normal control (NC) was treated with vehicle instead of test agents. Data indicate mean ± SD (*n* = 6). ^#^ *p* < 0.05 vs. NC. * *p* < 0.05 vs. TNBS.

The intrarectal injection of TNBS also caused colon shortening, increased myeloperoxidase, TNF-α, IL-1β, and IL-17 expression and NF-κB^+^CD11c^+^ cell population, and suppressed IL-10 and mucin-2 expression in the colon (Figure 6, Supplementary Figure S4). However, oral administration of LlLr, LlBl, LrBl, or LLB alleviated TNBS-induced colon shortening, decreased TNBS-induced myeloperoxidase, TNF-α, IL-1β, and IL-17 expression and NF-κB^+^CD11c^+^ cell population in the colon, and increased TNBS-suppressed IL-10 expression and mucin-2^+^ cell and claudin-1^+^ cell numbers, resulting in the amelioration of colitis. These combinations additively alleviated TNBS-induced colitis.

## 4. Discussion

The gut bidirectionally communicates with the brain through the nervous, endocrine, and immune systems [27,28]. The induction of gut microbiota dysbiosis by stressors such as pathogens and antibiotics triggers an inflammatory response, suppresses tight junction protein expression, and exacerbates systemic inflammation including neuroinflammation and colitis through an increased gut permeability, which easily translocate gut bacteria and their byproducts such as LPS into the body [9,25,29,30,31]. Bacterial LPS is a strong mediator of inflammation [9,32,33,34]. The translocation of LPS into the blood stimulates brain–blood barrier endothelial, leptomeningeal, and microglial cells, leading to psychiatric diseases with neuroinflammation [9,30,34]. 

We found that the intrarectal injection of TNBS in mice caused colonic inflammation and decreased the expression of claudin-1, a tight junction protein, and mucin-2, a member of mucin proteins, in the colon. Moreover, TNBS treatment increased LPS level in the blood and feces. These results suggest that TNBS treatment can induce LPS-absorbable colitis (a leaky gut). Treatment with TNBS increased cognitive impairment-like behaviors with neuroinflammation, as previously reported [9]. Moreover, TNBS increased hippocampal NF-κB^+^Iba1^+^ cell and LPS^+^Iba1+ cell numbers, while hippocampal BDNF^+^NeuN^+^ cell and claudin-5+ cell numbers decreased. Jang et al. reported that TNBS treatment impaired cognitive function and suppressed BDNF expression in the hippocampus [9]. Stressors including diets with high fat, antibiotics, immobilization stress, and TNBS increase gut bacterial LPS in the intestine [9,25,35,36,37]. Gut bacterial LPS induces gut inflammation, which increases gut permeability of microbial products such as LPS [25,37]. We also found that TNBS treatment increased fecal and blood LPS levels in mice and decreased the expression of tight junction protein claudin-1 in the colon and claudin-5 in the hippocampus. Exposure to LPS causes neuroinflammation and neurodegeneration in vivo [25,34]. These results suggest that TNBS treatment may cause cognitive impairment with neuroinflammation by inducing hippocampal NF-κB activation and suppressing LPS-hippocampal BDNF expression through an increase in the brain–blood barrier permeability of LPS. 

However, NK209, NK210, and NK219 singly or together suppressed TNBS-induced expression of TNF-α and IL-1β in the colon and hippocampus, while IL-10 expression increased. They increased TNBS-suppressed claudin-1 and mucin barrier protein mucin-2 expression in the colon and claudin-5 in the hippocampus, resulting in the attenuation of TNBS-induced colitis and neuroinflammation. These probiotics also decreased blood and fecal LPS levels. They also increased claudin-1 expression in TNBS-stimulated Caco-2 cells in vitro. These findings suggest that NK209, NK210, and NK219 may singly or together mitigate colitis and neuroinflammation by increasing mucin-2 and tight junction protein expression and decreasing fecal LPS production. 

Oral administration of NK209, NK210, and/or NK219 alleviated impaired cognitive behaviors in TNBS-induced colitis. They decreased NF-κB^+^Iba1^+^ cell and LPS^+^Iba1^+^ cell populations and increased BDNF expression and BDNF^+^NeuN^+^ cell population in the hippocampus. LPS production-inhibiting gut bacteria *Lactobacillus plantarum* NK151 and *Bifidobacterium longum* NK173 [38] alleviate cognitive impairment in mice. *Lactobacillus johnsonii* CJLJ103 mitigated scopolamine-induced cognitive impairment in mice by increasing NF-κB-involved BDNF expression [39]. ProBiotic-4, a probiotics mix, alleviated cognitive deficits in aged SAMP8 mice by suppressing TLR4-mediated NF-κB activation [40]. These findings suggest that NK209, NK210, and NK219 can singly or together alleviate cognitive impairment by increasing LPS-induced NF-κB activation-mediated BDNF expression. 

## 5. Conclusions

TNBS induced gut inflammation with increased LPS-permeable leaky gut and impaired cognitive function. NK209, NK210, NK219, and their combinations may alleviate cognitive impairment with systemic inflammation by suppressing the absorption of gut bacterial products including LPS into the blood through the suppression of gut bacterial LPS production and alleviation of a leaky gut by increasing gut tight junction proteins and mucin-2 expression.

## Data Availability

The datasets used and/or analyzed during the current study are available from the corresponding author on reasonable request.

## Supplementary Materials

The following supporting information can be downloaded at: https://www.mdpi.com/article/10.3390/nu14142975/s1. Figure S1: Effects of NK209 (Ll), NK210 (Lr), and NK219 (Bl) on NF-κB^+^Iba1^+^ (a), BDNF^+^NeuN^+^ (b), LPS^+^Iba1^+^ (c), and claudin (Cla)-5^+^ (d) cell populations in the hippocampus of mice with TNBS-impaired cognitive function. Figure S2: Effects of NK209 (Ll), NK210 (Lr), and NK219 (Bl) on NF-κB^+^CD11c^+^ (a), claudin (Cla)-1^+^ (b), and mucin (Muc)2^+^ (c) cell populations in the colon of mice with TNBS-induced colitis. Figure S3: The combined effects of NK209 (Ll), NK210 (Lr), and NK219 (Bl) on NF-κB^+^Iba1^+^ (a), BDNF^+^NeuN^+^ (b), LPS^+^Iba1^+^ (c), and claudin (Cla)-5^+^ (d) cell populations in the hippocampus of mice with TNBS-impaired cognitive function. Figure S4: The combined effects of NK209 (Ll), NK210 (Lr), and NK219 (Bl) on NF-κB^+^CD11c^+^ (a), claudin (Cla)-1^+^ (b), and mucin (Muc)2^+^ (c) cell populations in the colon of mice with TNBS-induced colitis.
