# Supplementary material for: Citation: Tight Junction Protein Expression-Inducing Probiotics Alleviate TNBS-Induced Cognitive Impairment with Colitis in Mice
Source: Nutrients. 2022 Jul 20;14(14):2975. doi: 10.3390/nu14142975 (PMC9317072; doi:10.3390/nu14142975)
Supplement: Supplementary file 1 [file nutrients-14-02975-s001.zip › nutrients-1767339-supplementary.pdf]

[Supplementary Materials]

Article

Tight junction protein expression-inducing probiotics alleviate TN BS-induced cognitive impairment with colitis in mice

Xiao-Yang Ma, Young-Hoo Son, Jong-Wook Yoo, Min-Kyung Joo, Dong-Hyun Kim*

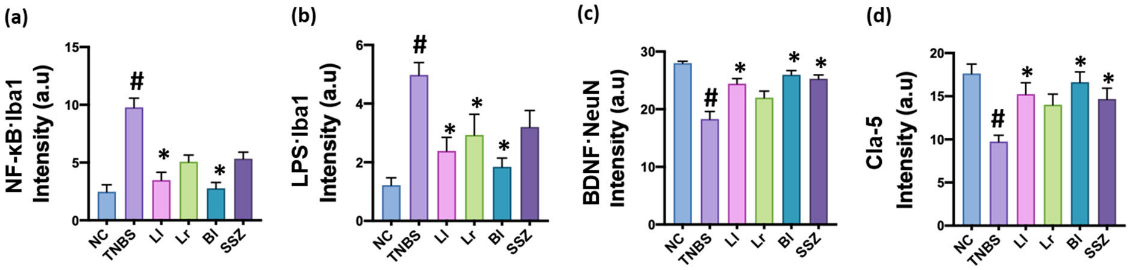

Figure S1. Effects of NK209 (Ll), NK210 (Lr), and NK219 (Bl) on NF-κB$^+$Iba1$^+$ (a), BDNF$^+$NeuN$^+$ (b), LPS$^+$Iba1$^+$ (c), claudin (Cla)-5$^+$ cell populations in the hippocampus of mice with TNBS-impaired cognitive function. The entire intensities of the single plane images were quantified by ImageJ software (github.com/imagej/imagej1). Data indicate mean ± SD (n = 8). #$p < 0.05$ vs. NC. *$p < 0.05$ vs. TNBS.

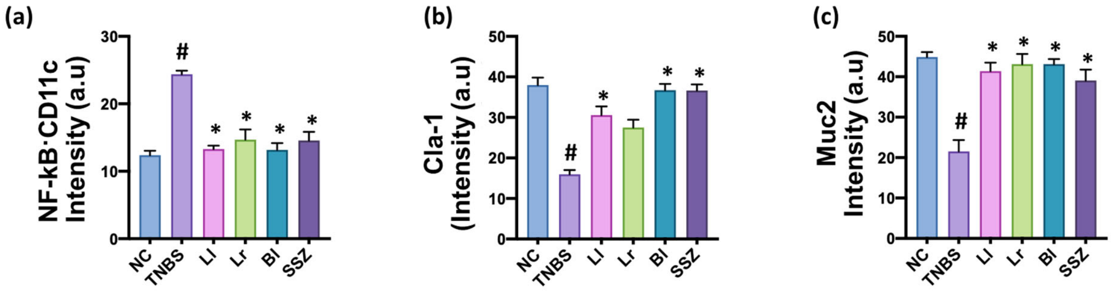

Figure S2 Effects of NK209 (Ll), NK210 (Lr), and NK219 (Bl) on NF-κB$^+$CD11c$^+$ (a), claudin (Cla)-1$^+$ (b), and mucin (Muc)2$^+$ (c) cell populations in the colon of mice with TNBS-induced colitis. The entire intensities of the single plane images were quantified by ImageJ software (github.com/imagej/imagej1). Data indicate mean ± SD (n = 8). #$p < 0.05$ vs. NC. *$p < 0.05$ vs. TNBS.

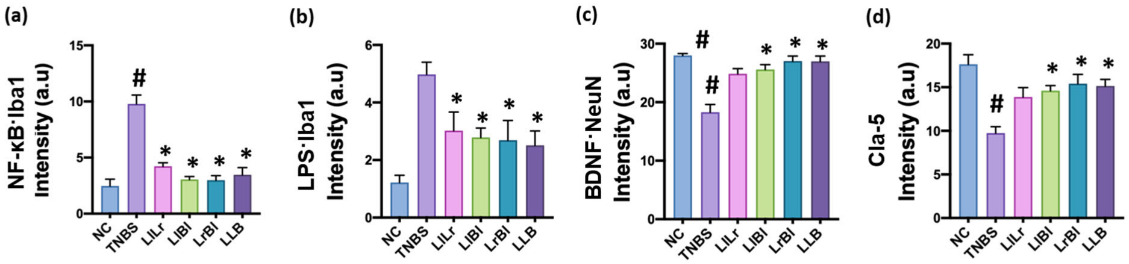

Figure S3. The combined effects of NK209 (Ll), NK210 (Lr), and NK219 (Bl) on NF-κB$^+$Iba1$^+$ (a), BDNF$^+$NeuN$^+$ (b), LPS$^+$Iba1$^+$ (c), claudin (Cla)-5$^+$ cell populations in the hippocampus of mice with TNBS-impaired cognitive function. The entire intensities of the single plane images were quantified by ImageJ software (github.com/imagej/imagej1). Data indicate mean ± SD (n = 8). #$p < 0.05$ vs. NC. *$p < 0.05$ vs. TNBS.

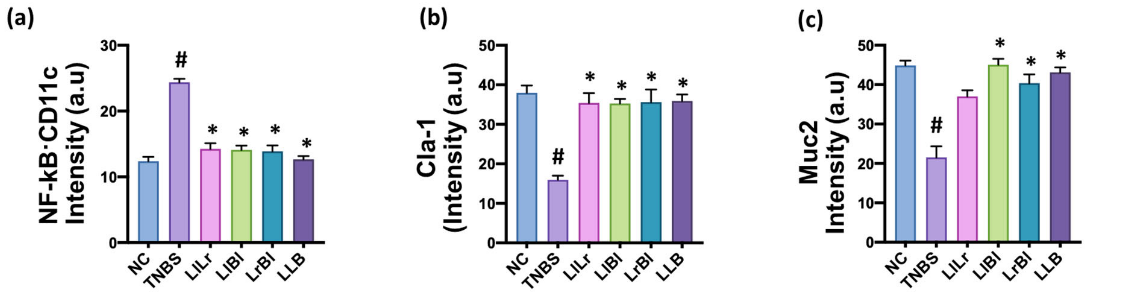

Figure S4. Figure 6. The combined effects of NK209 (Ll), NK210 (Lr), and NK219 (Bl) on NF-κB$^+$CD 11c$^+$ (a), claudin (Cla)-1$^+$ (b), and mucin (Muc)2$^+$ (c) cell populations in the colon of mice with TNBS-induced colitis. The entire intensities of the single plane images were quantified by ImageJ software (github.com/imagej/imagej1). Data indicate mean ± SD (n = 8). #$p < 0.05$ vs. NC. *$p < 0.05$ vs. TNBS.
